# Identification of mutations that alter the gating of the *Escherichia coli* mechanosensitive channel protein, MscK

**DOI:** 10.1111/j.1365-2958.2007.05672.x

**Published:** 2007-04-01

**Authors:** Chan Li, Michelle D Edwards, Hochterl Jeong, John Roth, Ian R Booth

**Affiliations:** 1 School of Medical Sciences, University of Aberdeen, Institute of Medical Sciences Foresterhill, Aberdeen AB25 2ZD, UK.; 2 College of Biological Sciences, Section of Microbiology, University of California Davis, CA 95616-5270, USA.

## Abstract

Mechanosensitive channels allow bacteria to survive rapid increases in turgor pressure. Substantial questions remain as to how these channels sense and respond to mechanical stress. Here we describe a set of mutants with alterations in their MscK channel protein. The mutants were detected fortuitously by their enhanced ability to modify the accumulation of quinolinic acid. Some amino acid changes lie in the putative pore region of MscK, but others affect sequences that lie amino-terminal to the domain aligning with MscS. We demonstrate that the alterations in MscK cause the channel to open more frequently in the absence of excessive mechanical stress. This is manifested in changes in sensitivity to external K^+^ by cells expressing the mutant proteins. Single-channel analysis highlighted a range of gating behaviours: activation at lower pressures than the wild type, inability to achieve the fully open state or a modified requirement for K^+^. Thus, the dominant uptake phenotype of these mutants may result from a defect in their ability to regulate the gating of MscK. The locations of the substituted residues suggest that the overall gating mechanism of MscK is comparable to that of MscS, but with subtleties introduced by the additional protein sequences in MscK.

## Introduction

Mechanosensitive (MS) channels play an important role in regulating turgor pressure in bacteria (Booth and Louis, 1999). When cells growing in hyperosmotic environments are shifted into hypoosmotic conditions (downshock), water flows through the cytoplasmic membrane causing a rapid increase in cell turgor pressure. MS channels in the membrane are gated by pressure such that they open at a level just below that which would cause significant damage to the cell wall (Levina *et al*., 1999). The non-specific release of solutes through the channels diminishes the driving force for water entry and thereby reduces turgor (Berrier *et al*., 1992). Four kinds of MS channel activities have been identified in *Escherichia coli* and are defined either by their electrical conductance or by other properties: MscL, large conductance (Sukharev *et al*., 1994); MscS, small conductance (Martinac *et al*., 1987); MscK, potassium-dependent small conductance (Levina *et al*., 1999; Li *et al*., 2002), and MscM, of mini conductance (Berrier *et al*., 1996). Conductance of the open state correlates with the pressure required to gate the channel, with MscM requiring least pressure and MscL the greatest. In addition to aiding survival of hypoosmotic shock, MS channels may play an important role during cell wall re-modelling upon entry to, and exit from, stationary phase (Stokes *et al*., 2003). These roles in cell physiology are underlined by the regulation of MS channel expression by osmotic stress and by stationary phase, with RpoS and other environmentally sensitive parameters controlling their transcription (Stokes *et al*., 2003; Schumann *et al*., 2004).

Considerable progress has been made on understanding the structural changes undertaken by MS channel proteins during the closed-to-open transition (Sukharev, 2000; Blount, 2003; Strop *et al*., 2003). In the open state, MscS and MscL create transient pores of ∼11–30 Å diameter (Sukharev, 2002), which must close sufficiently to prevent H^+^ from crossing the membrane (Levina *et al*., 1999). The crystal structures of MscS from *E. coli* and MscL from *Mycobacterium tuberculosis* have been solved (Chang *et al*., 1998; Bass *et al*., 2002). While the precise states represented by these structures is still subject to debate, models have been generated for the gating transition based on these structures and on complementary genetic, biochemical, electrophysiological and biophysical analyses (Blount *et al*., 1997; Moe *et al*., 1998; 2000; Sukharev *et al*., 1999; Perozo *et al*., 2002a, b; Edwards *et al*., 2005). In these models, both proteins have hydrophobic seals that block the channel pore in the closed state, but they differ in their mechanism of opening. MscL helices undergo a major change in tilt within the plane of the membrane to effect the closed-open transition (Sukharev *et al*., 2001a, b; Perozo *et al*., 2002a). In contrast, MscS helices rotate and slide across the surface presented by the adjacent helix, which is a more subtle structural alteration and correspondingly the pore created is smaller (Edwards *et al*., 2005). A further significant difference between the two types of channels is that MscS possesses a large cytoplasmically located vestibule perforated by seven lateral and one axial portal, which may act as pre-filters for solutes that will exit from the cell (Bass *et al*., 2002). It has been suggested that a smaller but equivalent structure may exist in MscL, but whereas this structure is static, there is evidence that changes in the MscS vestibule are dynamically coupled to the gating transition (Bass *et al*., 2002; Koprowski and Kubalski, 2003; Miller *et al*., 2003a).

**Fig. 1:**
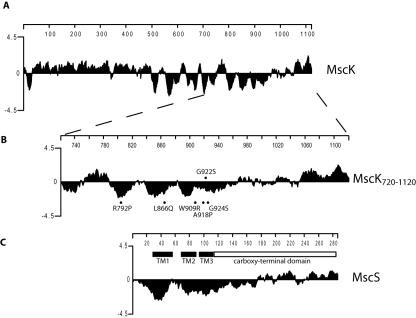
Organization of MscK and MscS. Hydrophobicity plots for *E. coli* MscK and MscS proteins, using the Kyte–Doolittle algorithm (Kyte and Doolittle, 1982) with a window of 19 residues, were created using the DNAstar suite of programs. A. Full-length MscK. B. The MscK region corresponding most closely to MscS, showing the positions of the different mutations tested in this study; those originally identified from *Salmonella* MscK (below line); and that from *E. coli* strain RQ2 (above line). C. MscS, showing the positions of the three transmembrane (TM) helices and the carboxy-terminal domain deduced from the crystal structure (Bass *et al*., 2002).

MscS is part of a very large family of membrane proteins, not all of which exhibit MS channel activity (M.D. Edwards and I.R. Booth, unpublished). Multiple homologues are present in many organisms, suggesting the possibility of alternative functions for some (Levina *et al*., 1999). The largest MscS homologue in *E. coli* is the MscK channel (previously, the structural gene has been called *aefA* and *kefA*; throughout this article we refer to the gene as *mscK* and the protein as MscK), which is one of the largest members of the MS channel family (1120 amino acids). MscK has 11 transmembrane spans with an N_OUT_–C_IN_ organization, based on alkaline phosphatase fusion analysis (McLaggan *et al*., 2002) (Fig. 1A). A cleavable amino-terminal signal sequence places the first ∼450-residue domain in the periplasm. This is followed by the membrane domain that appears to consist of two subdomains of seven and four spans; the smaller of these domains corresponds to the MscS membrane domain and is followed by the carboxy-terminal domain (∼190 residues). The last 257 amino acids of MscK exhibit significant sequence similarity to MscS (25% identity, 50% similarity; blast score 1e^−16^), with the region of similarity covering the region most clearly resolved in the crystal structure of MscS (Levina *et al*., 1999; Bass *et al*., 2002) (Fig. 1B and C). Electrophysiological analysis of MscK demonstrated that although the channel shares a similar conductance to MscS, its properties differ in several respects (Li *et al*., 2002). The MscK channel is less selective towards anions than MscS, is activated by slightly lower pressures and does not display the significant desensitization seen in MscS channels (Levina *et al*., 1999). Most significantly, however, activation of MscK in isolated patches requires both pressure and high concentrations of K^+^ on the periplasmic side (Li *et al*., 2002).

There have been only two chromosomal MscK mutants studied to date, a double-point mutant, *kefA2* (L565Q/G922S), and the null, created by integration of a drug resistance cassette (Levina *et al*., 1999; McLaggan *et al*., 2002). The *mscK* null mutant exhibits no strong growth phenotype alone, but leads to the complete loss of small MS channel activity in the presence of a *mscS* null mutation. The *kefA2* mutant allele in *E. coli* (strain RQ2) exhibits an unusual phenotype – sensitivity to high external K^+^ concentrations in the presence of compatible solutes (McLaggan *et al*., 2002). Under these growth conditions, bacteria normally replace internal K^+^ with betaine, which stimulates growth (Booth *et al*., 1988); in the mutant the exchange inhibits growth of the cells. Based on electrophysiological analysis, the mutant channel gated at lower pressures than its parent channel and no longer required high K^+^ concentrations for activation (Li *et al*., 2002). To date, genetic analysis of the structure and function of MscK has essentially been restricted to this single *E. coli* mutant, *kefA2*. This analysis would clearly benefit from availability of more mutant alleles.

This study reports the isolation of MscK mutants of *Salmonella typhimurium* as suppressers of a *nadB* mutation. The *nadB* gene encodes the first enzyme for the *de novo* synthesis of NAD but mutants at this locus can utilize external sources of pyridines as biosynthetic intermediates. Quinolinic acid (QA) can rescue this phenotype but only when it is provided at a high external concentration (10 mM), presumably because there is no specific mechanism for its transport. Genetic analysis identified mutations in *mscK* that facilitated uptake of QA, allowing *nadB* mutant cells to grow on low concentrations. The mutations found in *Salmonella* were introduced into the cloned *E. coli mscK* gene and their impact on cell physiology and electrophysiological signatures was determined. The two proteins are highly similar (89% identical, 95% similar by blast; Altschul *et al*., 1997), with most of the significant differences lying in the signal sequence, making the *E. coli* protein a good test-bed for analysis of the *Salmonella* mutations. We demonstrate that they cause the MscK channel to exhibit altered gating, such that the channels are frequently open in the absence of applied hypoosmotic stress. We show that the changes in cell physiology associated with the mutations are consistent with this modified gating. The locations of the substituted residues (Fig. 1B) suggest that the overall mechanism of pore formation in MscK is comparable to that of MscS but that the sequence differences generate distinct position-specific sensitivities to amino acid substitutions.

## Results

### Isolation of *mscK* alleles

From genetic screens for suppressers of a *nadB* mutation, common spontaneous mutants (*pnu*) arose that permitted growth of the *S. typhimurium nadB* mutant on 0.1 mM QA. Most of these mutants increased expression of the gamma amino butyrate permease (GabP), which can transport QA (H. Jeong and J. Roth, unpublished). However, one exceptional class proved to affect the *mscK* gene, suggesting that the altered channel protein allowed entry of QA.

Overall, 31 independent *pnu* mutants were isolated and assigned to groups pnuF*–I* by genetic mapping and sequencing (Jeong, 2003). The first characterized *pnu* mutation that later proved to affect the *mscK* gene was pnu*-189. Based on transductional linkage (41%) to this mutation, a Tn*10* insertion (*zba-3799::*Tn*10*dTc) was isolated and mapped near the *apt* locus. Six pnu* isolates were co-transducible with this element and were called pnuH* alleles. A previously isolated Tn*10*dTc isolate (*zbb-101::*Tn*10*dTc; strain TL1773), which maps close to *apt*, was transduced into pnuH* strains and was found to eliminate the capacity for growth on 0.1 mM QA. This insertion was found to be located between the +2235G and +2236T nucleotides of the *S. typhimurium mscK* gene, which encodes MscK, the potassium-dependent MS channel of small conductance (Levina *et al.*, 1999). Using *mscK*-specific primers, the individual point mutations were amplified and sequenced, revealing the affected residues to be R792P, L866Q, W909R, A918P and G924S (the R792P mutation was independently isolated twice) (Table 1). Of the five different pnuH* point mutations three affected the region equivalent to the pore-forming transmembrane helix (TM3) of the MscS channel in *E. coli* (Fig. 1B and C) (Levina *et al.*, 1999) and adjacent to the previously characterized dominant *kefA2* allele, G922S (McLaggan *et al.*, 2002). However, two mutations, R792P and L866Q, are situated amino-terminal to the pore region, with L866Q lying near the base of TM1 and R792P lying outside the sequences equivalent to MscS and thus defining a new region, specific to MscK, that affects channel behaviour.

**Table 1:** Sequence changes observed for pnuH* mutant alleles.

		Nucleotide sequence		
			
Strain	Genotype	Wild type	Mutant^a^	Amino acid change
TT16290	*pnuH183*	CTG	CAG	Leu_866_Gln
TT16294	*pnuH187*	CGT	CCT	Arg_792_Pro
TT16296	*pnuH189*	TGG	CGG	Trp_909_Arg
TT16297	*pnuH190*	CGT	CCT	Arg_792_Pro
TT16299	*pnuH192*	GGC	AGC	Gly_924_Ser
TT18216	*pnuH255*	GCG	CCG	Ala_918_Pro

a The underlined base indicates the change in the DNA sequence.

DNA sequence and consequent amino acid changes associated with the *pnu* mutations at the *mscK* locus that confer the ability to grow in the presence of low concentrations of quinolinic acid.

In the course of isolating Tn*10* insertions near the pnuH* gene, we discovered an unlinked insertion (*zac-3696::*Tn*10*dTc) that eliminated growth on 0.1 mM QA without altering the QA utilization phenotype of any other class of pnu* mutation. The insertion was mapped (at 2.8 min) far from the pnuH* region (11 min) and sequencing showed that it affected the *leuO* gene, which has been implicated as a stationary-phase global regulator (Ueguchi and Mizuno, 1993; Klauck *et al.*, 1997; Fang *et al.*, 2000; Fernandez-Mora *et al.*, 2004; Rodriguez-Morales *et al.*, 2006). This observation suggests a role for LeuO in regulating MscK expression in *Salmonella* and a possible role for expression of MscK in stationary-phase adaptation, similar to that proposed for the *E. coli* MS channels, MscS and MscL (Stokes *et al.*, 2003).

### Cloned *mscK* alleles do not rescue the *E. coli* chromosomal *kefA2* phenotype

**Fig. 2:**
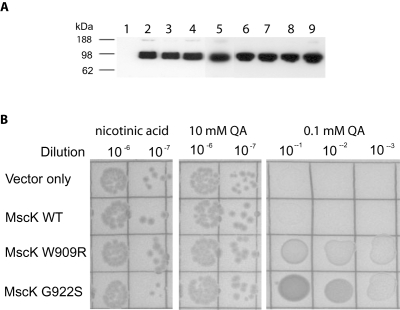
Expression of MscK mutant proteins and complementation of the *nadB* phenotype. A. Mutations were created using pTrcMscK as template and Quickchange (Stratagene) mutagenesis. Membrane preparations derived from MJF465 expressing wild-type MscK or MscK mutants were collected after induction with 0.3 mM IPTG for 30 min. Expression was detected by Western blot analysis using anti-His_6_ antibodies (Sigma). Lanes 1–9 correspond to: vector pTrc99A, wild-type MscK, L565Q, G922S, double mutant L565Q/G922S, W909R, L866Q, G924S and R792P respectively. B. *Salmonella typhimurium* strain TT22824 (*nadB499*::*mudJ*, *mscK267*::Tn*10*dTc) was transformed with plasmids as described in *Experimental procedures* and after overnight growth in minimal medium with nicotinic acid, the cultures were serially diluted onto agar plates containing either nicotinic acid (NA; 1 μg ml^−1^) or quinolinic acid (QA; 10 mM or 0.1 mM) and photographed after 24 h growth for NA and 10 mM QA and after 72 h for 0.1 mM QA.

To develop the analysis of the channels, the mutations observed in *Salmonella* were created in the *E. coli mscK* gene, using clones carrying the L565Q or G922S mutations as controls. The *mscK* genes were expressed from a moderate-copy-number plasmid under the control of the IPTG-inducible promoter, pTrc (see *Experimental procedures*). With the single exception of A918P, expression of the mutant proteins was not detrimental to growth and all of the proteins accumulated to similar levels in the membrane (Fig. 2A). The A918P mutant could only be created in a T7-based plasmid in a host lacking T7 polymerase, which is consistent with a strong gain-of-function (GOF) phenotype (data not shown) (Edwards *et al.*, 2005). The G922S mutant channel, which is a known GOF allele (Li *et al.*, 2002; McLaggan *et al.*, 2002), was tested for its ability to complement the *nadB* phenotype of *Salmonella* strain TT22824 (*nadB499*::*mudJ*, *mscK267*::Tn*10*dTc), as the selection of this mutation had a different basis (McLaggan *et al.*, 2002). Transformants carrying the W909R or wild-type *mscK* gene were used as controls, as the former, but not the latter confers the ability to grow on plates containing 0.1 mM QA (Fig. 2B). By this assay it was determined that the G922S allele (RQ2-derived) was able to complement the QA deficiency, which is consistent with the *mscK* mutations being effective through changes in channel gating. All transformants tested in this assay grew with either 10 mM QA or nicotinic acid (Fig. 2B).

**Fig. 3:**
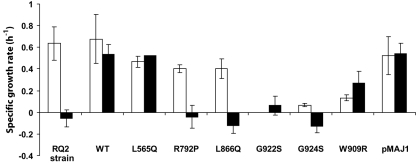
Effects of MscK mutations on growth of strain RQ2 in high-K^+^ or -Na^+^ medium in the presence of betaine. RQ2 cells expressing the MscK constructs were grown in K_20_ medium to exponential phase, diluted 20-fold into K_20_ containing either 0.6 M K^+^ or Na^+^ plus 50 μM IPTG and 1 mM betaine and the OD_650_ of the cultures measured every 30 min. Specific growth rates were calculated and are compared (mean ± SD) for growth under high-K^+^ (filled columns) or high-Na^+^ (open columns) conditions (*n* = 3 for each). RQ2 strain, *E. coli* mutant previously described (McLaggan *et al*., 2002); WT, pTrcMscK; L565Q, R792P, L866Q, G922S, G924S and W909R, mutations created in pTrcMscK as described in *Experimental procedures*; pMAJ1, *E. coli mscK* expressed from its own promoter (McLaggan *et al*., 2002). Negative values show slow progressive reduction in light scattering indicating a complete lack of growth caused by induction of the cloned *mscK* gene.

Strain RQ2 (*kefA2*; L565Q/G922S) fails to grow in high-K^+^ (0.6 M) medium in the presence of compatible solutes (McLaggan *et al.*, 2002); growth is restored by coexpressing a wild-type MscK channel. Strain RQ2 was transformed with the plasmids bearing the *E. coli mscK* gene mutated to express either the *Salmonella*-derived *mscK* mutations or the RQ2 mutations. Strains with these plasmids were grown to exponential phase in K_20_ and then diluted into 0.6 M salt containing 1 mM betaine (and IPTG) and growth followed over 5 h. Wild-type MscK and the L565Q mutant allowed normal growth of RQ2 in high-K^+^ medium in the presence of betaine (Fig. 3). However, mutants R792P, L866Q, W909R and G924S were either unable or poorly able to complement RQ2 (Fig. 3). As expected, the G922S mutant (Fig. 3) and the double mutant L565Q/G922S (equivalent to the chromosomal RQ2 mutations) were unable to complement the RQ2 phenotype (data not shown). GOF mutations favour the open conformation of the channel more than the wild-type protein, usually by altering the pressure sensitivity of gating (Blount *et al.*, 1996; Li *et al.*, 2002). The failure of the six mutant channels to restore growth of RQ2 in the high-K^+^ medium is consistent with the mutations causing altered gating of the MscK channel (Fig. 3).

**Fig. 4:**
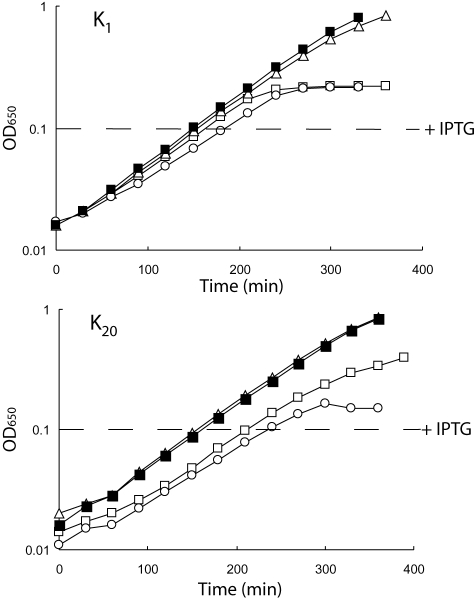
Effects of expression of MscK mutants on the growth of MJF465. MJF465 transformed with the MscK plasmids were grown overnight in K_1_ or K_20_ medium supplemented with ampicillin and then diluted 100-fold into K_1_ or K_20_ medium respectively. IPTG was added to a final concentration of 50 μM when the OD_650_ reached 0.1 and growth was followed. Representative growth curves are shown for cells grown in K_1_ medium (top) and cells grown in K_20_ medium (bottom). Dotted line indicates time of IPTG addition. Data are shown for pTrcMscK (closed squares), L866Q (open triangles), W909R (open squares) and G922S (open circles). Data for R792P were almost identical to those for L866Q and data for G924S were identical to those for G922S (and are omitted for clarity).

More subtle differences were illuminated by analysis of growth in high-Na^+^ medium, in the presence of betaine. The RQ2 strain grew well in this medium (as previously shown; McLaggan *et al.*, 2002) with a specific rate of 0.6 h^−1^ and expression of wild-type MscK from a plasmid did not significantly affect growth (Fig. 3). Several of the altered MscK channel proteins caused growth inhibition of strain RQ2 on Na^+^-based high-osmolarity medium containing betaine (Fig. 3). To clarify these effects the plasmids were introduced into *E. coli* strain MJF465 (MscL^–^, MscS^–^, MscK^–^) and growth was investigated in low-osmolarity medium containing different K^+^ concentrations (K_1_ and K_20_). The growth rate in such media is affected by the frequency of channel gating that can lead either to loss of K^+^ homeostasis or to depolarization of the membrane. In K_1_ medium, prior to addition of IPTG, the low-level expression of plasmids carrying the G992S mutation (i.e. G922S and L565Q/G922S) led to significant growth inhibition, whereas the other plasmids caused little or no change to the growth rate (Fig. 4). Within 60 min of addition of IPTG (50 μM) growth was completely inhibited for cells expressing the G922S, L565Q/G922S, G924S and W909R mutations, whereas growth of cells expressing the wild-type, L565Q, L866Q, or R792P MscK was not affected (Fig. 4). Similar growth patterns were observed in K_20_ medium, with the exception that growth of cells induced for expression of W909R appeared significantly better than in K_1_ medium (Fig. 4). The mutations causing the most severe depression of growth are those that are in the equivalent positions to key residues in the TM3 pore-forming region of MscS–G922 in MscK is equivalent to A106 in MscS, and G924 is equivalent to G108 (Edwards *et al.*, 2005), whereas W909 aligns with T93 that lies in the extended chain region of the upper pore of MscS (Miller *et al.*, 2003b).

### MscK GOF mutants restore K^+^ uptake to a transport-deficient mutant

**Fig. 5:**
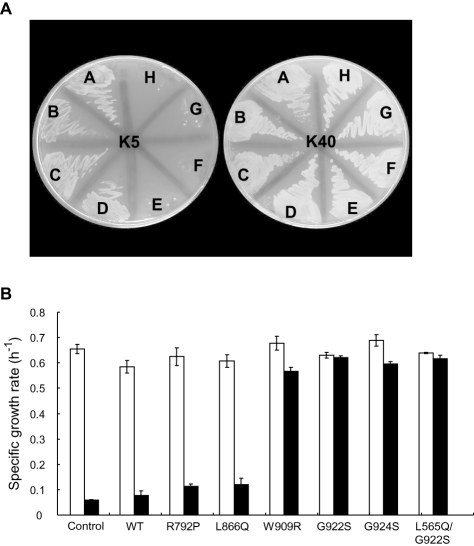
Assessment of the opening frequency of the MscK mutant channels using complementation of the K^+^ transport defect of a triple K^+^-uptake mutant. A. Single colonies of transformants of MJF603 (TK2309, *mscK*::Kan) carrying each mutant plasmid were streaked onto minimal medium plates containing 5–115 mM K^+^, incubated at 37°C for 24 h and then photographed. Data are shown for plates containing either 5 mM K^+^ (left) or 40 mM K^+^ (right): A, double mutant L565Q/G922S; B, G924S; C, G922S; D, W909R; E, L866Q; F, R792P; G, L565Q; and H, wild-type MscK. B. Minimal medium was inoculated with a culture that had been grown to exponential phase in K_40_, such that the final concentration of K^+^ was either 9 mM (closed bars) or 40 mM (open bars) and the cells were grown in a shaking incubator at 37°C (*n* = 3). The OD_650_ was measured at intervals and the specific growth rate determined from log-linear plots. Control, parent strain without any plasmid; WT, pTrcMscK; other mutations, created in pTrcMscK as described in *Experimental procedures*.

Suppression of the *E. coli* growth defect associated with loss of the major transport systems Trk, Kup and Kdp has frequently been used to detect either channel activity or K^+^ transport activity associated with cloned genes (Kim *et al*., 1998; Uozumi, 2001; Irizarry *et al*., 2002; Buurman *et al*., 2004; Kraegeloh *et al*., 2005). To investigate the potential of the mutant MscK proteins to activate spontaneously at low turgor pressure, a *mscK* null derivative of the *E. coli* strain TK2309 (MJF603, see *Experimental procedures*), which lacks the major K^+^ uptake systems Trk, Kup and Kdp, was created by transduction. This strain grows normally in media containing 20 mM or more K^+^ but fails to grow with only 5 mM K^+^ present (Fig. 5). To test the capacity of MscK to allow K^+^ transport, the mutated constructs were introduced into MJF603 and the transformants were streaked on minimal medium plates containing 5, 20, 40 or 115 mM K^+^. All transformants grew well on plates containing either 20 mM (data not shown) or 40 mM K^+^ (Fig. 5A) but the transformants expressing the G922S, G924S or L565Q/G922S mutations were slightly impaired in their growth on 115 mM K^+^ (data not shown). The wild-type MscK channel was unable to restore growth on plates containing 5 mM K^+^, but transformants expressing G922S, G924S, W909R or L565Q/G922S mutant channels grew normally at this low concentration (Fig. 5A). Similar data were obtained in liquid media, with growth being observed in K_9_ minimal medium in strain MJF603 expressing the same group of mutations but no rescue associated with the expression of the wild-type, R792P or L866Q MscK proteins (Fig. 5B). Therefore, it appears likely that G922S, G924S and W909R mutant channels gate sufficiently frequently at low osmolarity to allow rapid K^+^ entry. The data obtained at 115 mM K^+^ suggests that the channels bearing G922S or G924S mutations are gating sufficiently frequently to slow growth under these conditions, possibly by causing significant depolarization of the cytoplasmic membrane.

### MscK pore mutations exhibit altered gating properties

The properties of the MscK channels were analysed directly by patch clamp on isolated patches derived from appropriate transformants of strain MJF429 (MscS^–^, MscK^–^). Constitutive expression of MscL in this strain allows comparison of the pressure required to activate the mutant MscK channels with the parent channel (Li *et al*., 2002). The analysis was performed at pH 7, in the presence of 200 mM K^+^, which has been observed to be required on the periplasmic side of the membrane for gating of MscK in excised membrane patches (Li *et al*., 2002). Unless otherwise stated, recordings were made from cell preparations grown in the absence of IPTG and therefore represent the basal expression of the channel from the *trc* promoter, as described previously (Miller *et al*., 2003b). In a previous study we reported that MscK activity expressed from the chromosomal copy of the gene was only observed in ∼30% of recordings in which MscL was detected (Li *et al*., 2002). Using the cloned gene the frequency of patches that showed both MscK and MscL activity was routinely observed to be between 30% and 80%, indicating that functional MscK channels are still in low abundance in membrane patches relative to the native profusion of MscS and MscL.

**Fig. 6:**
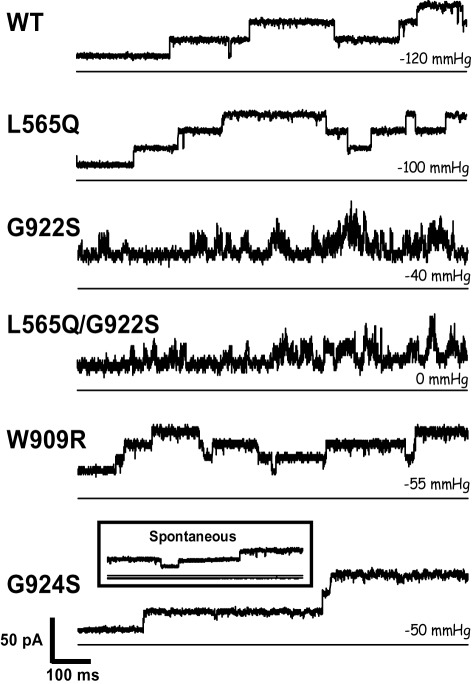
Electrophysiological recordings of MscK channels. The wild-type and mutant MscK constructs were expressed in *E. coli* strain MJF429 and protoplasts prepared, as described in *Experimental procedures*. Inside-out excised patches were used to record single-channel activity. Representative traces are shown for all proteins that stably expressed in the membrane and gated with reproducible characteristics. Note that channels bearing the G922S mutation were unable to sustain the open conformation, thus exhibiting the appearance of flickery activity, and some mutant channels opened spontaneously, examples shown for the double mutant L565Q/G922S and the single mutant G924S (inset) where activity has occurred in the absence of pressure.

MscK channel activity was characterized by stepwise openings and closures with an average open dwell time of ∼100 ms, which is similar to MscS channels (Edwards *et al*., 2005). The pressure required to activate MscL is used as a reference for the gating pressure for MscK (P_L_:P_K_) and MscS (P_L_:P_S_). We observed that the pressure needed to activate MscK was reproducibly lower (P_L_:P_K_∼1.87 ± 0.07; *n* = 7) than the activation pressure for MscS (P_L_:P_S_∼1.59 ± 0.03) and the conductance was also smaller than for MscS channels: 880 ± 50 pS (*n* = 9) for MscK compared with ∼1250 pS for MscS (Edwards *et al*., 2005). The RQ2 strain has two mutations in the *mscK* gene, L565Q and G922S. We have previously shown that it is the G922S mutation that is likely to be the major contributor to the phenotype (McLaggan *et al*., 2002) and we confirmed this here by electrophysiology. MscK L565Q behaves exactly like the wild-type MscK channel (P_L_:P_K_∼1.78 ± 0.05; *n* = 8), consistent with the physiological properties of this mutant (Fig. 6). The G922S and the L565Q/G922S mutant channels displayed frequent openings with lower thresholds of activation (P_L_:P_K_∼2.40 ± 0.10 and ∼2.65 ± 0.11, respectively; *n* = 6 for each), which is consistent with the analysis of the equivalent chromosomally encoded channel in strain RQ2 (Li *et al*., 2002), and some spontaneous activity was also observed for the double mutant. However, both G922S-containing mutant channels failed to achieve a stable open state before closing, producing a flickery appearance of channel activity in recordings and prohibiting accurate measurements of the conductance of the fully open state (Fig. 6). These data confirm the importance of the G922S mutation for the phenotype of the RQ2 MscK channels.

The properties of the other mutant channels lead to two well-defined groups: channels that exhibit openings and closures similar to the parent, but are altered in their pressure sensitivity, and channels that exhibit extremely unstable activity. The former class, including W909R and G924S, display regular openings and closures, but gate at lower pressures than wild-type MscK [P_L_:P_K_∼2.47 ± 0.12 and ∼4.38 ± 0.66, respectively, for W909R (*n* = 3) and G924S (*n* = 7)] and both showed some spontaneous gating in the absence of applied pressure (Fig. 6). For these mutants the conductance was similar to the parent, consistent with the mutations affecting the sensitivity to pressure rather than the structure of the conducting pore. The A918P mutation caused channel openings to be unstable and exhibit a much reduced conductance (data not shown). Similar properties are associated with a mutation at the equivalent position in MscS (A102P; M.D. Edwards, W. Bartlett and I.R. Booth, unpublished) (Miller *et al*., 2003b). Two mutant channels, R792P and L866Q, were not easily detected in protoplast patches and when pressure-induced activity was observed it exhibited unusual behaviour. These channels displayed irregular openings with different conductance values; even for the same patch, repeat applications of pressure could result in variable responses from these mutant channels (see Fig. S1). Essentially, the R792P channels sometimes exhibited frequent closures in contrast with the wild-type channels, but other times they opened to a stable wild type-like state. Similar behaviour was observed for the L866Q channel, but with more extreme variability. Finally, attempts to measure the threshold pressures of these inconsistent openings showed that these mutant channels usually required greater pressure to open than the wild-type MscK under patch clamp conditions (with approximate pressure ratios – due to the variable nature – ranging from ∼1 to 1.5).

Li *et al*. (2002) established that the chromosomally mutated MscK protein in strain RQ2 could gate in response to pressure in the absence of high K^+^ concentrations. Here we tested the ability of the cloned MscK mutant channels to open in recording solutions containing Na^+^ in place of K^+^. As expected, mutation G922S generated flickery channel openings in the absence of K^+^ however, these channels required greater pressure to open in Na^+^ than in K^+^ (P_L_:P_K_∼1.33 ± 0.16 in Na^+^; *n* = 4). Although W909R MscK channels open with a lower pressure than wild-type MscK in the presence of K^+^, these mutant channels did not readily gate in Na^+^ buffer and if observed, it was at exceptionally high pressures (close to that sufficient to gate MscL or rupture the membrane patch). In contrast, mutant channels containing the G924S substitution readily opened in the absence of K^+^, although activation under these conditions always required pressure and at a level greater than when K^+^ was present (P_L_:P_K_∼1.88 ± 0.16; *n* = 8). Attempts to record from cells expressing either R792P or L866Q mutant channels in Na^+^ solutions produced similar irregular, unstable activity to that seen in K^+^ buffer. Thus, it appears that Ser substitution of the two Gly residues in the predicted MscK pore creates channels with a strong GOF phenotype where K^+^ is no longer necessary for gating but is essential for activation at lower pressures.

**Fig. 7:**
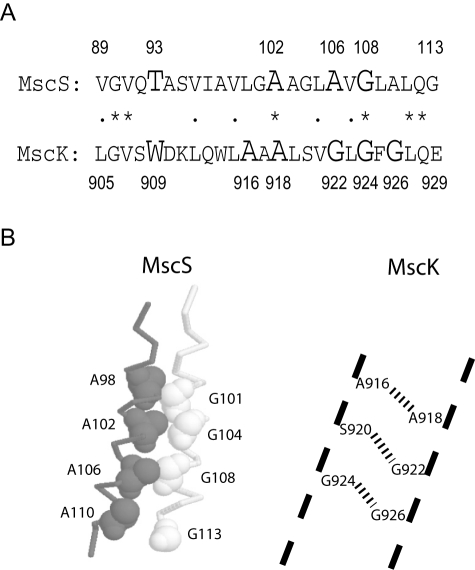
Alignment of the putative pore region of MscK with the pore region of MscS. A. Identical and conserved residues are marked with asterisks and dots respectively. Residues in MscK that have led to gain-of-function phenotypes when mutated are indicated in larger font. Similarly, equivalent residues in MscS that have yielded gain-of-function channels (Miller *et al*., 2003b; Edwards *et al*., 2005) are also highlighted in larger font. B. Two helices from MscS (left) and MscK (right) are depicted to indicate the interface residue pairing for MscS (derived from the crystal structure) (Bass *et al*., 2002) and suggested interface pairing for MscK. The pairings for MscK have been created by analogy with the MscS structure on the assumptions that (i) MscK forms a heptamer and (ii) the helix crossing angles are not changed relative to MscS.

**Fig. 8:**
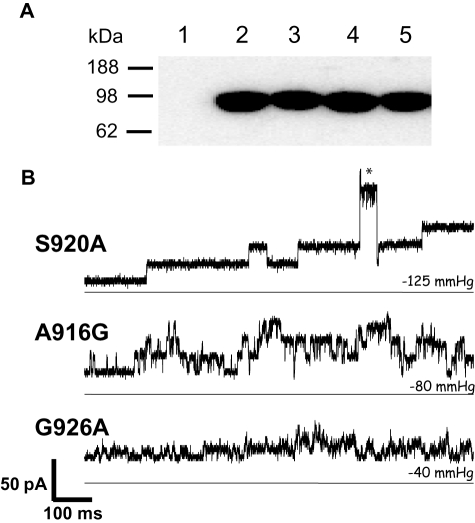
Expression and electrophysiological recordings of the engineered pore mutants. A. MJF465 cells expressing either wild-type or the mutant MscK plasmids were induced with 0.3 mM IPTG for 30 min and harvested for membrane preparation. Expression was detected by Western blot using anti-His_6_ antibodies (Sigma). Lanes 1–5 correspond to: vector pTrc99A, wild-type MscK, A916G, S920A and G926A respectively. B. Representative single-channel recordings of the three mutant channels after expression in strain MJF429 and preparation of protoplasts. Note that S920A MscK channels opened at pressures close to those required to open MscL channels (see text) and as such, a MscL single-channel opening is present in this trace (denoted by an asterisk).

The data presented above are consistent with the gating mechanism being similar to that of MscS despite the sequence differences in this region (only 20% identity and 40% similarity in the 21 residues between Thr93 and Gly113 of MscS; see Fig. 7A). To extend this analysis we used our previous investigations of MscS gating to select amino acid changes for which a predicted phenotype might be made. MscS exhibits a conserved packing interface in the TM3 pore-lining helices that is constructed from three Ala–Gly pairs. Disruption of an Ala–Gly packing interaction by introduction of a Gly residue in place of the Ala decreased the pressure required to gate the channel. In contrast, substitutions of Gly with Ala increased threshold pressure for activation. Introduction of Ser residues modified the gating pressure and channel characteristics in a position-specific manner (Edwards *et al*., 2005). Molecular modelling (Kim *et al*., 2003; 2004; Edwards *et al*., 2005) of the MscK putative pore-lining helices identified the following packing motif: W_914_xAxAxSxGLGxGLQ_928_ (S. Kim, C. Li and I.R. Booth, unpublished) that has the potential to create packing surfaces between adjacent pore-lining helices that comprise Ala_916_–Ala_918_, Ser_920_–Gly_922_ and Gly_924_–Gly_926_ interactions (see Fig. 7B), which is significantly different from that observed in MscS. To help understand the relationship between pore sequence and packing we created A916G, S920A and G926A with the prediction that these changes would decrease (A916G) and increase (S920A and G926A), respectively, the pressure for gating MscK. All three mutants were readily created and the proteins expressed to levels equivalent to the wild-type channel (Fig. 8A). As predicted, the A916G channel gated at lower pressures than the wild type (P_L_:P_K_∼2.55 ± 0.21; *n* = 7). These mutant channels exhibited a significant shortening of the peak mean open dwell time (∼7 ms compared with ∼100 ms for wild-type channels; data not shown); however, they were able to attain the fully open state (Fig. 8B). MscK S920A channels were essentially wild type in their open-state conductance (Fig. 8B) and open dwell time (∼250 ms; not shown) but required more pressure to open them (P_L_:P_K_∼1.17 ± 0.02; *n* = 10). Consistent with their electrophysiological character, the expression of the A916G mutant channels inhibited growth in high-osmolarity medium (both high Na^+^ and high K^+^ in the presence of betaine) and did not complement the mutant phenotype of the RQ2 strain. However, expression of the S920A channel did not impair growth at high osmolarity and efficiently complemented RQ2, consistent with the loss of function associated with this allele (data not shown). The most surprising result was obtained with G926A, which was expected to exhibit a channel that was more difficult to open. These mutant channels gated with low pressures (P_L_:P_K_∼2.65 ± 0.17; *n* = 6), although they rarely opened to the full extent observed when wild-type channels gate (Fig. 8B). In this respect, G926A behaved in a similar manner to G922S and it was not surprising therefore that expression of this mutant channel inhibited growth at high osmolarity in a K^+^-independent manner and failed to complement strain RQ2 (data not shown). These data, while confirming the trends observed in mutational analysis of MscS (Edwards *et al*., 2005), show subtle differences in the pore sequence requirements for normal gating of the MscK channel.

## Discussion

There is a great multiplicity of MS channel proteins and their homologues in enteric bacteria, with at least seven genes identified and a further activity, MscM, for which no gene product has yet been assigned (Pivetti *et al*., 2003). In addition, it is known that transport systems can acquire point mutations that alter their specificity leading to channel-like properties (Buurman *et al*., 2004) and still other channels have been identified but have no assigned physiological role (Milkman, 1994; Kuo *et al*., 2003). In a wide genetic screen for mutants of *S. typhimurium* with improved ability to take up QA, 31 suppresser mutations were isolated, of which six mapped to *mscK* (one allele being isolated twice), but none were found in other putative or confirmed genes for other MS channels (including *mscS* or *mscL*). The electrophysiological and growth phenotypes conferred by the mutations when transferred to the *E. coli* MscK protein provide a probable explanation for the improved QA uptake based on the aberrant gating of the channels. This is particularly true for the three mutations in the putative pore-lining helix, where we have demonstrated that the MscK alleles have altered pressure sensitivity, such that the threshold for activation is lowered. Spontaneous activation of the channel may create a new pathway for entry of QA, as is observed for K^+^ in the triple K^+^ transport mutant (Fig. 5). However, in the presence of high K^+^ concentrations, frequent channel openings will depolarize the membrane, as has been noted for MscL mutants (Buurman *et al*., 2004). Thus, the MscK mutant channels may either provide the route for QA entry or potentiate accumulation of QA by lowering the membrane potential. Among the other classes of alleles suppressing the high QA-dependence phenotype of the *nadB* mutant strain were mutations that inactivated a drug export system and others that increased expression of a catabolite repressed GabP permease, which normally transports gamma amino butyrate (H. Jeong and J. Roth, unpublished). These data suggest that both altered uptake and diminished efflux of QA can contribute to the observed reduction in concentration dependence of growth.

The only previously recorded point mutations affecting the chromosomal copy of MS channels were MscK *kefA2* (strain RQ2) (McLaggan *et al.*, 2002) and a MscL N15D mutation (Buurman *et al.*, 2004). A partial explanation for this observation can be derived from a consideration of the properties of RQ2. Here we have demonstrated that when the G922S mutation was introduced onto a plasmid-borne *mscK* gene a much more obvious phenotype was observed than in the previous study where only the chromosomal locus was investigated. The RQ2 strain (L565Q/G922S on the chromosome) can grow in high-Na^+^ medium in the presence of betaine (McLaggan *et al.*, 2002); however, expression of G922S from a plasmid inhibited growth in the same conditions (Fig. 3). In contrast, increased expression of wild-type MscK was without effect on growth in high-Na^+^ medium. Similarly, increased expression of the G922S mutant channel inhibited growth on minimal medium containing either 1 mM or 20 mM K^+^ (Fig. 4), conditions under which the chromosomal mutation was without observable effect (McLaggan *et al.*, 2002). Protein abundance, which is affected by gene copy number and control over expression, affects the observed phenotype for these channel mutations. A similar conclusion can be drawn from recent analyses of MscL, where an N15D mutation was isolated in the *E. coli* chromosomal gene as a suppresser of the K^+^ transport defect in the triple K^+^ transport null strain (Buurman *et al.*, 2004) but the same mutation caused severe growth inhibition when expressed from a plasmid (Ou *et al.*, 1998). This may help to explain the paucity of chromosomal mutations that alter channel gating and the failure to retrieve MscS GOF mutants from generalized screens with expression from high-copy-number plasmids (Okada *et al.*, 2002).

### Strong GOF mutations reside in the pore-lining helix

Three of the five mutations isolated in a screen for improved QA import affected the putative pore region of MscK (W909R, A918P and G924S), which suggests comparable gating mechanisms for MscS and MscK, despite the dissimilarities in the pore helix sequences. Whereas both proteins are predicted to have two rings of hydrophobic residues creating the hydrophobic seal, the tight Ala–Gly packing, which was seen as the basis for the helix rotation and tilt model of MscS gating (Edwards *et al*., 2005), is not conserved in MscK (Fig. 7). Molecular modelling of MscK pore helices indicates the potential for both hexameric and heptameric organization (S. Kim *et al.*, unpublished), which is consistent with the known potential of sm domains (the domain that lies just below the predicted pore region) to exist in both hexameric and heptameric assemblies (Mura *et al*., 2003). Such packing would create Ala–Ala, Ser–Gly and Gly–Gly interfaces, which have been shown to modify the pressure sensitivity and open dwell time properties of MscS channels (Edwards *et al*., 2005). The clash of Ala with Ala increased the pressure to gate MscS, whereas creating a Gly–Gly interface reduced the pressure needed. Introduction of Ser residues opposite Gly had position-specific effects, but usually lowered the pressure required to gate the channel. Two of the pore mutations engineered specifically in this study obeyed the predictions from the MscS model for packing-residue interactions (Edwards *et al*., 2005); A916G mutants (creating Gly–Ala from Ala–Ala) gate at lower pressure and S920A mutants (creating Ala–Gly from Ser–Gly) require higher pressure. Similarly, the behaviour of the MscK G924S mutant channel, selected from the screen for ability to use low QA concentrations, was not unexpected as the introduction of Ser residues into the pore-lining helix of MscS generally caused GOF. Indeed, the strongest phenotypes in the MscK mutant channels are associated with introduction of either Ser or Pro residues that have the most potential to modify the helix properties. However, as with MscS, the actual position of the Ser residue is critical to the impact on channel function. Both G924S and G922S proteins gate at low pressure, but whereas G924S channels can achieve a stable open state, the G922S channels cannot (this may be due to the G922S channels collapsing to a closed state before reaching the fully open state or it may be that they spend a significantly reduced time in the open state, where the temporal resolution of our recording system precludes absolute measurement of peak amplitude). It is notable that these two mutations are the only ones to confer significant independence of K^+^ at the periplasmic face when gating, which may be due to these proteins forming conformations that already reduce the energy barrier for gating such that pressure is now a sufficient stimulus in the absence of K^+^ (and accordingly, less pressure is required for transition to the open state in the presence of the activating ion).

The MscK and MscS proteins exhibit significant sequence similarity in the regions flanking the putative pore-lining helix. A larger region of lower similarity extending back towards the amino-terminus of MscS TM1 can be aligned but with decreased statistics, indicating that TM1 and TM2 have diverged significantly during the separate evolution of MscK and MscS from some distant common ancestor. Indeed, hydrophobicity and alkaline phosphatase analysis of MscK would support the channel-forming domain having four transmembrane helices rather than the three in MscS (Fig. 1) with a further seven TM domains located amino-terminal to the pore-forming domain (McLaggan *et al*., 2002). Consistent with this proposal is the observation that between the seventh and eighth TM domains there is an extended hydrophilic stretch of sequence (residues 751–780; rriawrrala rrqnlvkega egaeppeept) that resembles a flexible linker. Many larger MscS homologues have at least four TM domains with a probable N_IN_–C_IN_ conformation, suggesting that the four-TM span model might be a frequent structure. One of the GOF alleles reported here, R792P, maps to the eighth TM span of MscK, which would correspond to the first helix of a four-TM channel-forming domain. As the only basic residue at the junction between TM8 and the putative linker, it is conceivable that the loss of the R792 charge at this position alters the anchoring of the helix, which would be consistent with the inability to obtain stable channel recordings in patch clamp analysis. Equally, introduction of a proline-induced turn might alter the trajectory of the TM span; however, this region is very rich in residues that are turn-generating as the sequence QVNQQT immediately precedes R792. While there are uncertainties about the mechanism by which the phenotype of the R792P mutation arises, the altered character of the channel points to the importance of this region for the function of MscK.

A second mutation also identifies a further region of importance for gating and/or stability of MscK. When modelled onto the MscS structure, the L866Q mutation lies close to the base of TM1 in a region that is moderately conserved between the two proteins. In the MscS structure this region is described as an α-helical turn, rather than a precise continuation of the TM1 helix. The potential for disruption in the equivalent MscK TM span is even greater as this region is even more enriched in helix-breaking residues than MscS. We have previously identified this region as critical for gating of MscS (Miller *et al*., 2003b) and the discovery that, in MscK, the introduction of another helix-breaking residue alters gating of the channel adds to the growing importance of this segment of the protein. However, it is notable that this residue lies close to the lipid–headgroup interface of the membrane and the introduction of a potential hydrogen-bonding donor at this position in place of Leu may change the protein fold leading to the observed changes in activity (Yoshimura *et al*., 2004; Nomura *et al*., 2006).

Finally, the discovery of MscK alleles as suppressers of QA dependence and their identification of altering channel gating properties led us to engineer other mutations in the putative pore region. The selected residues fitted with the model that we have previously advanced for paired residues in TM3 helices of MscS with the single exception of G926A. This was found to create a channel that gates at low pressure. The paradox is that this mutation, which from our predictions reprises in MscK the Gly–Ala matching seen in MscS and removes a Gly–Gly pairing, would be expected to predispose the channel to gate at higher pressure (Edwards *et al*., 2005). This raises the intriguing possibility that the MscK channel pore-lining helices adopt a different crossing angle from those observed in the crystal structure of MscS reflecting the significantly altered pattern of residues in the putative pore-lining helix of MscK. Structural analysis of this channel is underway and should reveal such changes and point the way to different structural algorithms for within the family of MS channels of the MscS/MscK class.

## Experimental procedures

### Media

Luria–Bertani medium (LB) contained: tryptone, 10 g; yeast extract, 5 g; and NaCl, 5 g, per litre. The minimal medium is denoted by K_*x*_, where ‘*x*’ is the mM K^+^ concentration (Epstein and Davies, 1970). K_115_ consisted of: K_2_HPO_4_, 46 mM; KH_2_PO_4_, 23 mM; (NH_4_)_2_SO_4_, 8 mM; MgSO_4_, 0.4 mM; FeSO_4_, 6 μM; sodium citrate, 1 mM; thiamine hydrochloride, 1 mg l^−1^; and glucose, 0.2% (w/v). For K_0_ medium, equimolar sodium phosphate replaced potassium phosphate. K_1_ was prepared by adding 1 M KCl to K_0_ (1 ml l^−1^). Mixing suitable proportions of K_0_ and K_115_ or addition of appropriate volumes of 1 M KCl to K_0_ was used to create medium with intermediate K^+^ concentrations (McLaggan *et al*., 2002). High-Na^+^ medium and high-K^+^ medium are defined as K_20_ supplemented with either 0.6 M NaCl or KCl respectively. E medium (Vogel and Bonner, 1956) consisted of (per litre): MgSO_4_·7H_2_O, 0.2 g; citric acid, 1.85 g; K_2_HPO_4_, 10 g; and NaNH_4_HPO_4_·4H_2_O, 3.5 g, pH 7, supplemented with 0.2% (w/v) glucose. Further supplementation with 1 μg ml^−1^ nicotinic acid or 10 or 0.1 mM quinolinic acid (final concentrations) was conducted as required. In all media, ampicillin was used at 50 μg ml^−1^ when required and all plates contained 1.4% (w/v) agar.

### Strains and plasmids

The initial mutant analysis was carried out in derivatives of *Salmonella enterica* serovar Typhimurium strain LT2. The parent strain for the hunt was TT10738 (*nadB499*::MudJ). Transposition-defective Tn*10* derivatives (TN*10*dTc) were used as described previously (Way *et al*., 1984; Elliott and Roth, 1988). Strain TL1773 (*zbb-101::*Tn*10*dTc) was the generous gift of Laszlo Csonka. *E. coli* strain MJF465 (*mscL*^–^*mscS*^–^*mscK*^–^) was used for channel protein expression and physiological analysis. JM109 was used as cloning host and strain RQ2 was used for complementation experiments (strain genotypes described previously; McLaggan *et al*., 2002). *E. coli* MJF603 (TK2309, *mscK::*Kan) was created by transduction of strain TK2309 (F^–^*thi, rha, lacZ, nagA, tr kDa*1*, trkA*405*, kdp*::Tn*10*) to Kan^R^ using a P1 phage lysate generated from strain MJF379 (Levina *et al*., 1999). *S. typhimurium* TT22824 (*nadB499*::*mudJ*, *mscK267*::Tn*10*dTc) was used to assess the physiology of cells expressing the mutant MscK channels. Vector pTrc99A was purchased from Pharmacia and plasmid pNL1 consists of the vector pET21b (Pharmacia) carrying the *mscK* gene with a His_6_-tag on the carboxy-terminal end and two point mutations (N468K R469D), as described previously (McLaggan *et al*., 2002).

### Transduction with phage P22

The high-frequency generalized transducing phage P22 HT105/1 *int-201* was used for all transductional crosses (Schmieger, 1971) as described previously (Elliott and Roth, 1988; Grose *et al*., 2005).

### Subcloning of *mscK* and mutation creation

The plasmid pTrcMscK was made by subcloning *mscK* with six histidines on its carboxy-terminus from pNL1 (McLaggan *et al*., 2002) into the vector pTrc99A, using primer pair 5′-GCGCGAGCTCATGACTATGTTCCAGTATTAC-3′ and 5′-GCGCGTCGACTCAGTGGTGGTGGTGGTGGTG-3′, creating SacI and SalI sites on the 5′ and 3′ ends of *mscK* respectively (indicated by underlines). Starting with pTrcMscK as template, site-directed mutagenesis was performed using the Stratagene Quickchange protocol to create all mutant plasmids and the primers for each mutation are listed in Table 2. All mutant plasmids were sequenced on both strands twice, using the BIG-DYE reaction mix (Amersham) as instructed by the supplier.

**Table 2:** Primers used to create mutations in pTrcMscK.

Mutation	Primers
L565Q	5′-CTGCCGGTGTGCCTGATTATTCTCGCGGTTGGCCTG-3′
	5′-CTGCCGGTGTGCCAAATAATATTAGCGGTTGGCCTG-3′
R792P	5′-CCAGCAGACGCTACCTATTACCATGTTGC-3′
	5′-GCAACATGGTAATAGGTAGCGTCTGCTGG-3′
L866Q	5′-CGCAACCTGCCTGGCCAACTGGAAGTGCTGG-3′
	5′-CCAGCACTTCCAGTTGGCCAGGCAGGTTGCG-3′
W909R	5′-CGCTGGGCGTCTCGAGGGATAAACTCC-3′
	5′-GGAGTTTATCCCTCGAGACGCCCAGCG-3′
A918P	5′-CCAGTGGCTGGCCGCAGCATTATCCGTAGG-3′
	5′-CCTACGGATAATGCTGCGGCCAGCCACTGG-3′
G922S	5′-GCATTATCCGTATCCTTAGGTTTTGGTTTAC-3′
	5′-GTAAACCAAAACCTAAGGATACGGATAATGC-3′
G924S	5′-GCATTATCCGTAGGGCTCGGTTTTGGTTTACAAG-3′
	5′-CTTGTAAACCAAAACCGAGCCCTACGGATAATGC-3′

### Membrane preparations and Western blotting

The MscK plasmids were transformed into MJF465 cells, grown in LB to mid-exponential phase and induced with 0.3 mM IPTG for 30 min before harvesting. Membrane protein samples were collected as previously described (Towbin *et al*., 1979; Miller *et al*., 2003a, b). Briefly, cells were re-suspended in phosphate-buffered saline containing: 137 mM NaCl, 2.7 mM KCl, 10 mM NaH_2_PO_4_ and 1.4 mM K_2_HPO_4_, pH 7.4, with Complete™ EDTA-free protease inhibitors (Roche Diagnostics). Cells were lysed by French press (18 000 psi) and membrane preparations collected by differential centrifugation. Protein concentration in membrane samples was assayed by the Folin–Ciocalteau method (Lowry *et al*., 1951). Nitrocellulose blots containing the separated proteins were probed with anti-His_6_ antibodies (mouse IgG2a isotype; Sigma).

### Complementation of strain RQ2

RQ2 cells carrying MscK constructs were grown overnight in K_20_ medium with ampicillin and the following morning diluted 20-fold into fresh pre-warmed K_20_ medium supplemented with ampicillin and grown at 37°C with shaking (300 r.p.m.) to OD_650_ ∼ 0.4. The culture was then diluted 20-fold into K_20_ medium containing 0.6 M NaCl or KCl with 1 mM betaine, 50 μg ml^−1^ ampicillin and 50 μM IPTG and the OD_650_ of the culture was measured every 30 min. Specific growth rates were calculated from three separate experiments for each plasmid tested.

### Physiological assays of channel properties

(i) The MscK constructs were transformed into strain MJF465 and grown overnight in K_1_ or K_20_ minimal medium containing ampicillin. The next morning the cultures were diluted 100-fold into media containing the same concentration of K^+^ and grown at 37°C with shaking. Once an OD_650_ of 0.1 was reached, IPTG was added to give a final concentration of 50 μM and growth at 37°C was recorded every 30 min.

(ii) The MscK plasmids were transformed into strain MJF603. Fresh transformants were streaked onto minimal medium agar plates containing 5, 20, 40 or 115 mM K^+^ (agar was washed thoroughly with distilled water before making 5 mM plates). After incubation for 24 h at 37°C, the appearance of the growth zones was recorded by photography. To assess the specific growth rates of the transformants, a single colony was inoculated into minimal medium containing 40 mM K^+^ and grown to stationary phase overnight in a shaking incubator at 37°C. The overnight cultures were diluted into K_40_ and grown to OD_650_ ∼ 0.4, then cultures were diluted 10-fold into fresh pre-warmed growth medium containing either 5 mM or 40 mM K^+^ (final K^+^ concentration 9 mM and 40 mM respectively) and incubated with shaking at 37°C. Samples were taken at regular intervals and the OD_650_ was recorded. The specific growth rates were calculated from log-linear plots and the data for three experiments used to calculate mean and standard deviation.

(iii) Also, a selection of plasmids were transformed into *Salmonella* strain TT22824 and cultures were grown overnight in E medium supplemented with ampicillin, glucose and 1 μg ml^−1^ nicotinic acid (NA). The following day they were adjusted to the same OD_650_, washed and serially diluted using E medium without supplements. Aliquots (5 μl) were spotted onto E medium plates supplemented with glucose, ampicillin and either NA (1 μg ml^−1^) or quinolinic acid (QA; 10 or 0.1 mM). The plates were placed at 37°C and progress of colony growth was monitored.

### Electrophysiological assays

Patch clamp recordings (Hamill *et al*., 1981) were conducted as described previously (Li *et al*., 2002) using strain MJF429. Briefly, excised, inside-out patches were analysed at room temperature in symmetrical solutions containing (unless otherwise stated) 200 mM KCl, 90 mM MgCl_2_, 10 mM CaCl_2_ and 5 mM HEPES buffer at pH 7. For experiments testing K^+^ dependence, 200 mM NaCl replaced KCl. The giant protoplasts were generally prepared using cells that had not been induced for expression of the MscK plasmids as the basal level of expression from the cloned genes usually resulted in sufficient numbers of channels per patch. However, for Na^+^ buffer assays, induction with 1 mM IPTG (15–60 min) was used for some mutants to increase the probability of channels being present in any one patch. For these assays, an equal number of protoplasts were also assessed in K^+^ solutions to confirm the viability of each preparation. All data were acquired at a holding potential of 20 mV (pipette positive) and a sampling rate of 50 kHz with 5 kHz filtration, using an AxoPatch 200B amplifier in conjunction with Axoscope software PClamp9 (Axon). The pressure threshold for activation of the MscK channels, with respect to the activation threshold of MscL in any one patch (P_L_:P_K_), was determined as described previously for MscS channel pressure ratios (Blount *et al*., 1996; 1997; Ou *et al*., 1998). Dwell time and conductance analyses were undertaken using all point histograms. All measurements have been conducted on patches derived from at least two protoplast preparations and pressure ratios are given as mean ± SEM.

### Materials

LB medium components were purchased from Oxoid and minimal medium components, as well as all other chemicals, were obtained from Sigma or BDH. Restriction enzymes were from Roche or Promega. Pre-cast Novex polyacrylamide gels were obtained from Invitrogen-Life Technologies and the Supersignal Dura substrate came from Perbio.

## Supplementary material

The following supplementary material is available for this article online:

Fig. S1 R792P and L866Q MscK mutant channels exhibit irregular activities. Three excerpts from electrophysiological traces are shown as examples of the unusual behaviour of mutant MscK channels containing R792P or L866Q substitutions. The two upper panels for each mutant are traces from sequential pressure trials for that patch - note the short dwell time nature of opening and general increase in conductance from the first pressure application to the next (in both cases). The lower panel for each mutant comes from a different protoplast patch and depicts examples where openings were more wild type-like in their character, although they required MscL levels of gating pressure to open.

This material is available as part of the online article from http://www.blackwell-synergy.com
